# Application of quality indicators and critical lessons learned assessment as a research approach for the evaluation of rescue missions during terrorist attacks

**DOI:** 10.1038/s41598-024-76267-3

**Published:** 2024-10-23

**Authors:** Thomas Wurmb, Sebastian Kurz, Gerhard Schwarzmann, Herbert Trautner, Uwe Kinstle, Ulrich Wagenhäuser, Florian Koch, Markus Münch, Patrick Meybohm, Maximilian Kippnich

**Affiliations:** 1 Department of Anaesthesiology, Intensive Care, Emergency and Pain Medicine, Section Emergency and Disaster Medicine, University Hospital Würzburg, Oberdürrbacherstrasse 6, 97080 Würzburg, Germany; 2 Department of Anaesthesiology, Intensive Care, Emergency and Pain Medicine, Section Emergency and Disaster Medicine, University Hospital Würzburg, Würzburg, Germany; 3 University Hospital of Würzburg, Würzburg, Germany; 4 Johanniter Rescue Emergency Services of Wuerzburg, Würzburg, Germany; 5 Bavarian State Police, Lower Franconia Police Headquarters, Wuerzburg, Germany; 6 Fire and Rescue Integrated Control Centre, Wuerzburg, Germany; 7 Emergency Pastoral Care in the Diocese of Würzburg, Wuerzburg, Germany

**Keywords:** Terrorism, Lessons learned, Disaster response, Emergency planning, Health care, Health policy

## Abstract

In Wuerzburg, Germany, a terrorist attack and a killing rampage occurred five years apart (2016 and 2021). Following a structured evaluation of the rescue mission in 2016, a bundle of quality indicators and ten “lessons learned” were defined. Aim of the presented study was to compare the two rescue missions and to critically review the lessons learned from 2016 for their implementation and feasibility. An interdisciplinary and inter-professional group of experts analyzed the data using predefined quality indicators. All lessons defined in 2016 were critically reviewed and qualified as either lessons learned or lessons identified. While seven out of ten lessons were successfully implemented after 2016 (lessons learned), three lessons didn´t work and were recategorized as lessons identified (communication, zoning and the mutual exchange of different tactical approaches). Our results demonstrate that the conclusions drawn in 2016 have helped to improve the performance of the rescue forces in 2021. In addition, the identified lessons are now the basis for further improving emergency and disaster preparedness. It is important to understand, that the process of preparedness improvement is not completed with the definition of lessons identified. These must first be integrated into response plans and then trained intensively. A lesson identified only becomes a lesson learned once it has been successfully applied.

## Introduction

Terrorism and rampage are permanent threats, thus building up resilient emergency preparedness is a major issue. Even if the war in Ukraine and the near east crisis are currently the key issue, the challenges posed by terrorism and rampage need to be considered worldwide. In the last 10 years, there were many publications about the development and adaption of medical response strategies to terroristic attacks^1–10^. Stopping the dying as well as the killing was identified as an important principle^6^. The issue of triage and zoning was pointed out by several authors to be a key element of successful on scene management^6^. Other important key lessons were the management of uncertainties, of victims, of teams and communication^11^. Carli et al. published identified problems and mitigation measures after the Paris terror attack in 2015. The bullet points were the handling of war weapon injuries, the reaction to chemical weapons, the assault on hospitals, care under fire and psychological emergency care provision. This explains the special challenges for rescue forces during these operations. Unfamiliar injury patterns, operating under constant threat, direct and intense cooperation with police forces, difficult zoning and high psychological stress. Moreover, experts clearly stated the need for systematic evaluations and systematic reports after terror attacks. This will allow for the description, assessment and comparison of civil emergency rescue operations during terrorist attacks and they may serve as a very important basis in order to define and communicate the lessons learned^12^.

Schorscher et al. conducted a systematic review in order to identify lessons learned after terror attacks from 2001 to 2017 wordwide^13^ and described a brad pattern of lessons learned that was unchanged over the entire study period. The most important recurrent categories were tactics, communication, preparedness, training, triage, patient flow and multi-disciplinary cooperation. The authors concluded that the knowledge gained was often not put into practice and that the so-called lessons learned were in fact lessons identified. The lessons learned process, taken from "The NATO Lessons Learned Handbook" by the Joint Analysis and Lessons Learned Centre, is defined as follows: "Lesson learned (LL)" is the end point of a three-step process. The first step is to draw lessons from experience and to analyze and generalize them. The result of this first step is the "Lesson identified (LI)". The second step is to assign an appropriate action to the LI, such as adapting a recommendation for action and training the staff who will apply it. The third step consists of "closing the learning circle", on the one hand checking whether the change is being applied and on the other hand evaluating whether the desired improvement is occurring. If the change is applied and an improvement occurs as a result, this is the "lesson learned”^14^.

**Fig. 1:**
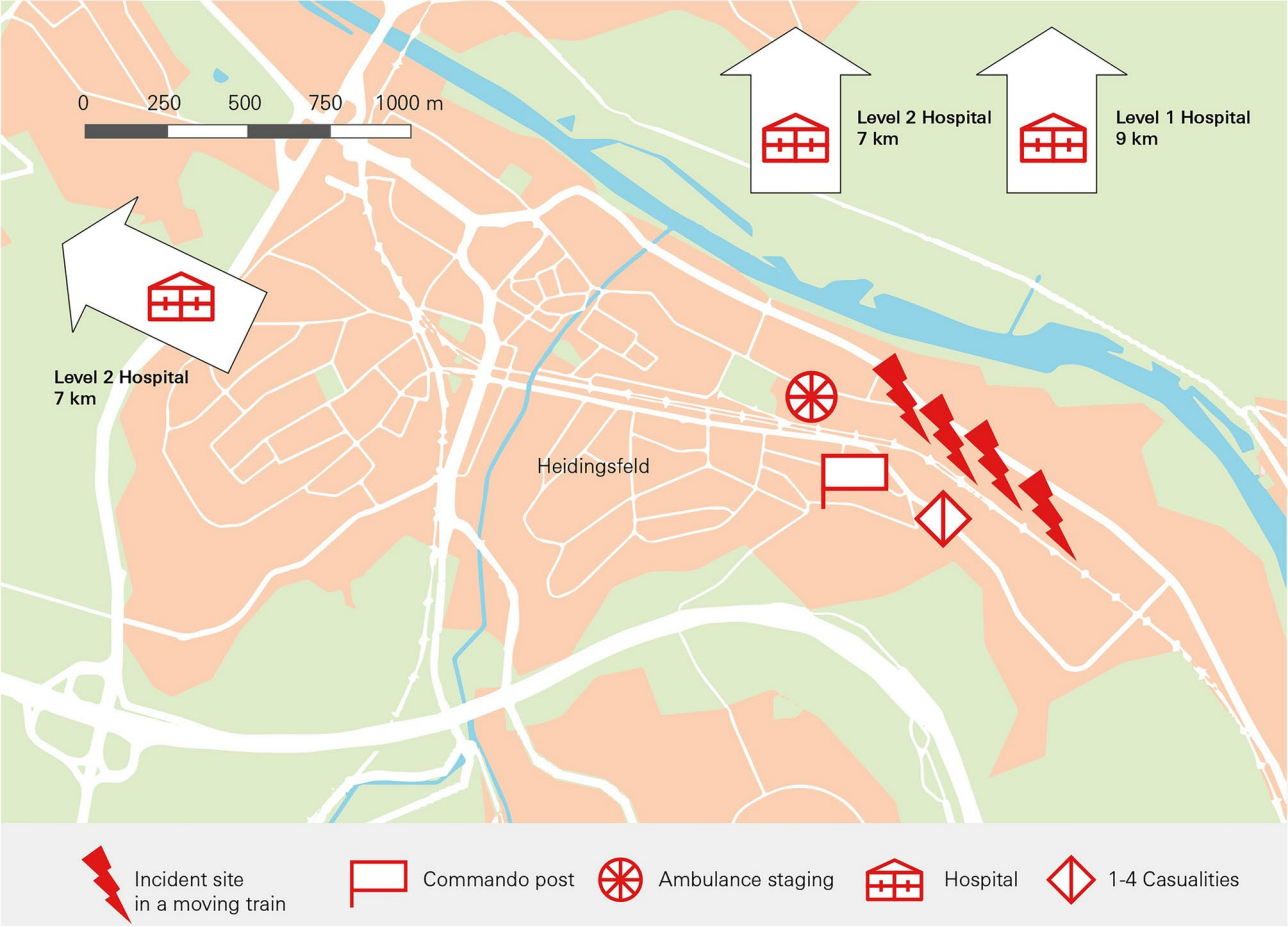
Incident site 2016.

**Fig. 2:**
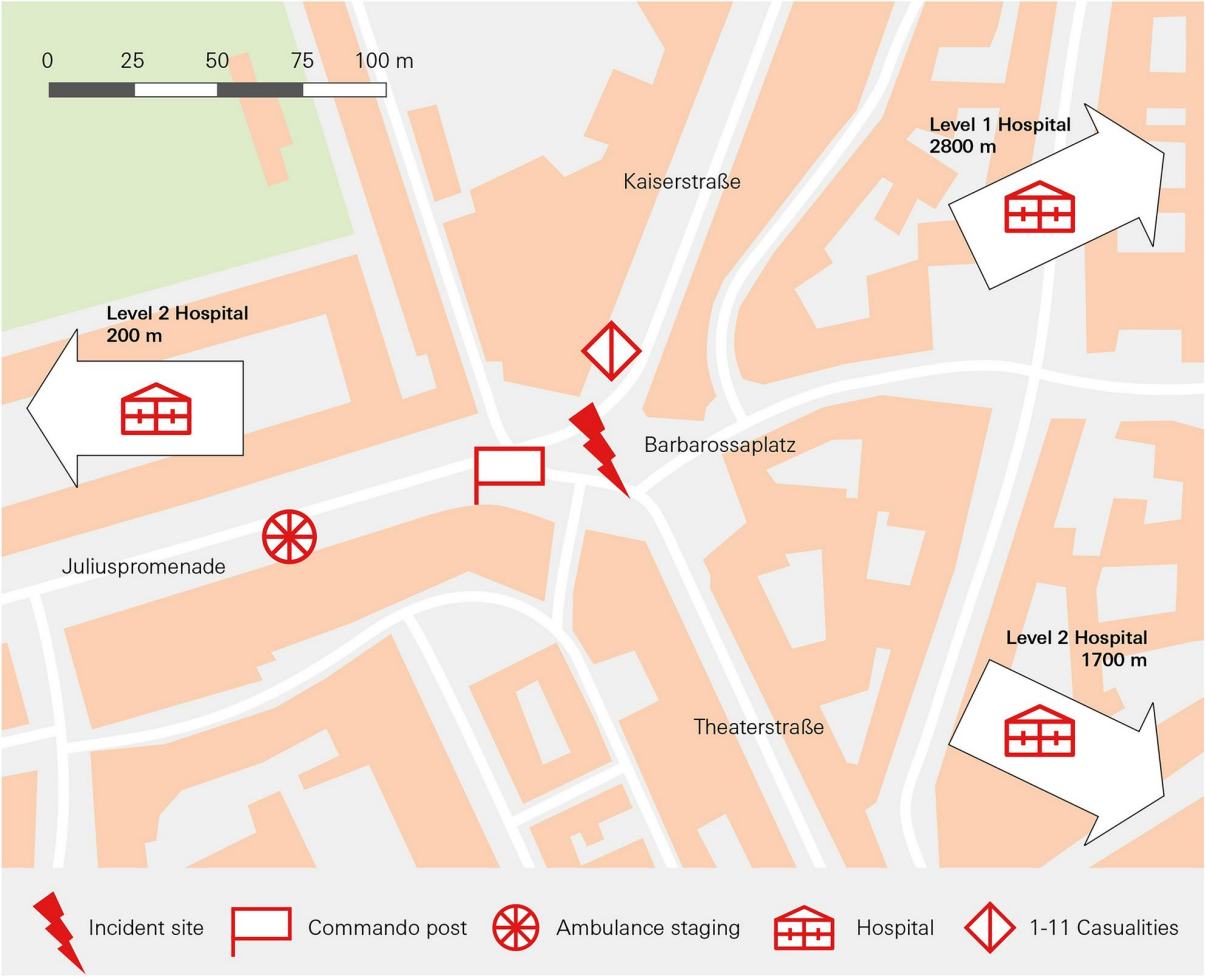
Incindent site 2021.

Würzburg, Germany was the scene of a terrorist attack in 2016 in which four people were seriously injured with an axe and a knife (Fig. 1). The rescue operation was analyzed using dedicated quality criteria, lessons learned were defined^5,13,15^ and the response plans were adapted to improve resilient emergency preparedness. Two important changes since 2016 are the change in operational tactics (clear up the scene immediately) and the expanded medical equipment for terrorist attacks. Five years later (2021), another rampage attack with a knife occurred in Würzburg, in which three people were killed and nine people were seriously injured (Fig. 2).

In this article, we compared both incidents and evaluated whether the findings described as lessons learned in 2016 have been successfully implemented in the response plans before the attack in 2021.

## Methods

According to the NATO lessons learned process an expert panel conducted a structured evaluation process. The project was presented to the local ethic committee of the University Würzburg and ethical approval was waived (Reference: 20180817 01).

The expert panel included leading representatives of the University Hospital Würzburg, the Emergency Medical Services (emergency physicians and paramedics), the police forces, the office for Emergency Medical Services and fire brigade alerting, the government of Lower Franconia, and the emergency pastoral care. The expert group was led by two renowned scientists whose field of research is emergency and disaster medicine. The other medical members all have academic qualifications and are regularly involved in scientific projects. The members of the rescue services are all in leading positions with higher-level tasks in rescue management. The police officers were also graduates with management responsibilities in all areas of police work. All members of the expert group were already involved in the scientific evaluation of the 2016 operation, and most of the experts were also on site as commanders during both operations.

All experts were members of the study team. There were no experiments or procedures including human participants.

Based on the 158 quality indicators identified in the 2016 evaluation^15^, anonymous data derived from records of the dispatch center, from the mission data of the rescue services, from general surveys and from the reports of the emergency services, were collected for the 2021 attack and entered in clustered tables.

**Fig. 3:**
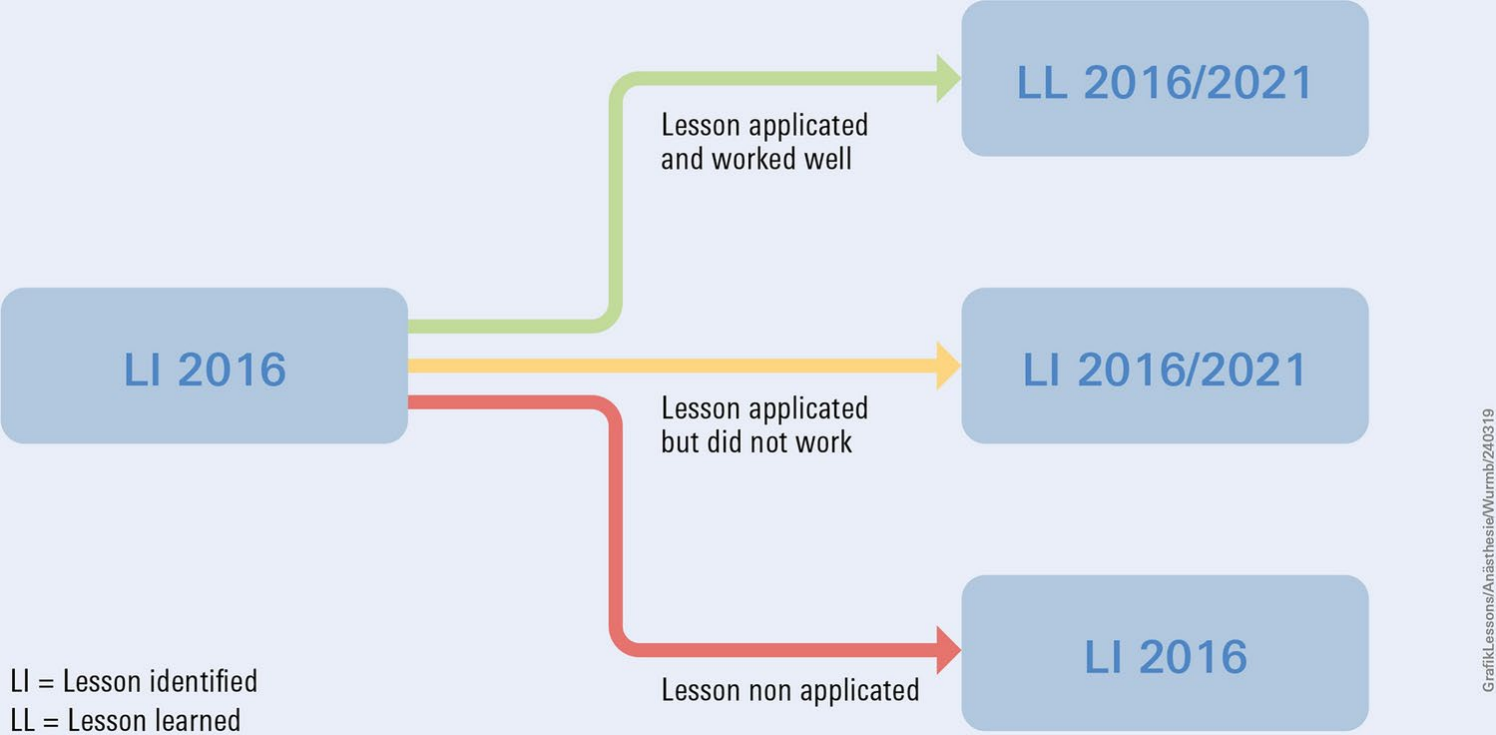
Flowchart of the comparison of lessons learned.

In order to follow the NATO lessons learned process, the findings from 2016, described as lessons learned (LL 2016), were reset to the status lesson identified (LI 2016). In a next step, each of these 10 lessons was reviewed to determine whether or not they had been properly implemented and applied in the 2021 rescue mission. If implementation was successful, it was defined as a lesson learned (LL 2016/2021). If the LI 2016 was not relevant for the mission, it remained at LI 2016 status. If the LI 2016 was applied but did not have the intended effect during the mission, it was defined as LI 2016/2021 (Fig. 3). If there were new findings in the 2021 evaluation they were classified as LI 2021. These definitions were made in a group-centered consensus process. Disagreements were discussed and either resolved or included as such in the results. (Fig  3)

## Results

A total of five meetings of the expert group were held in the period 2021–2023. Relevant descriptive data of the two rescue missions are shown in Tables 1, 2, 3, 4, 5, 6, 7.

**Table 1:** Incident characteristics.

General characteristics	Incident 2016	Incident 2021
Date	2016.07.18	2016.06.25
Weekday and time of day	Monday 09:14 pm	Friday 05:02 pm
Weather (rain, snow etc.)	20 °C, clowdy dry	18 °C sunny
Place of incident—rural area or city?	City of Würzburg (130.000 inhabitants) – Local Train	City of Würzburg (130.000 inhabitants)—City Center
Type of attack	One offender acting with an axe and a knife	One offender acting with a knife
Number of hospitals in the area (radius 50 km)	16	16
Number of local trauma centres?	4	4
Number of regional trauma centres?	3	3
Number of national trauma centres?	1	1
Is there a written concept for Mass Casualty Incidents (MCI) for the emergency medical services in place?	*yes*	yes
Is there a written and coherent concept for dealing with life threatening incidents/ rescue missions?	*yes*	yes
Total number of patients	8	11
Number of patients classified as T1/RED in medical triage	4	6
Number of patients classified as T2/YELLOW in medical triage	1	0
Number of patients classified as T3/GREEN in medical triage	1	2
Number of patients classified as T4/BLACK in medical triage	1	3
Number of hospitalised patients	6	8
Number of patients deceased at place of incident	1 (the offender)	3
Number of uninjured people who have been affected	15	30

**Table 2:** Mission related data.

Mission related data	Data 2016	Data 2021
At what time was it noticed that this was a life threatening incident for the rescue services? (Δt from first emergency call to notification of life threatening situation)	09:20 pm Δt: 6 min	05:04 pm Δt: 2 min
Who has first notified the incident (police, rescue headquarter or operational forces on scene)?	Police headquarter	Police headquarter
*Δ t from notification until communication between rescue headquarter and police headquarter*	*Δt: 0 min*	*Δt: 0 min*
*Δ t from alarm until successful notification of all operational forces about life threatening situation*	*Δt: 0 min*	*Δt: 0 min*
Use of weapons (knife etc.)?	Knife and axe	knife
Use of explosive agents?	no	no
Abuse of vehicles (truck/car)?	no	no
Chemical, biological, radio-nuclear threats (CBRN)	no	no
Any other imminent danger?	no	no
Combination of static and dynamic situation?	no	
Multisite attack?	no	no

**Table 3:** Communication.

Communication	Incident 2016	Incident 2021
Have there been a previously defined communication infrastructure between the emergency medical rescue headquarter and the police headquarter?	no	yes
Which communication system was used?	Radio and telephone	Radio and telephone
Has a communication plan been established before the mission started?	no	yes
Was the plan applied?		partly
*Have there been a regular and structured re-evaluation of the situation for the incident commander in chief throughout the different mission phases (continuous report flux)*	*no*	*Yes*
If so, have the results been recorded in a timely and structured method (mission diary/ map of incident site)?	no	yes
*Have an infrastructure for communication between medical services, police and fire services been established?*	*no*	*yes*
Did police, medical services and fire services communicate regularly and effectively with each other?	Yes: the subsection commanders No: The incident commanders in chief	*Due to high information load it was not possible to share information in an effective way*

**Table 4:** Triage.

Triage	Incident 2016	Incident 2021
Has a triage algorithm been used?	No specific algorithm was used	yes
If so, which triage algorithm was used?	none	mSTaRT
Δ t from arrival of first rescue services on scene until the start of triage?	Unknown	Immediately after arrival
Who was responsible for the triage?	Emergency medical services	First arriving ambulance
Have life saving measures been delivered during the first triage cycle?	yes	yes, control of bleeding
Δ t from arrival of first rescue services on scene and communication of primary triage result to the rescue headquarter	Δ t: 8 min	First triage result was displayed very early by a witnessing emergency physician
Have patients been treated according to triage priorities?	yes	yes
Have patients been allocated to hospital according to triage priority?	yes	yes

*mSTaRT* modified simple triage and rapid treatment.

**Table 5:** Casualty care.

Casualty care	Incident 2016	Incident 2021
Has medical care been based on damage control principles?	No, there was complete resuscitative care provided	yes
Has a stepwise care provision according to the different sectors been used (unsafe sector—principles of “Care under fire”; partly safe sector—principles of “Tactical Field Care”; safe sector – “Tactical Evacuation Care”?	No, the medical care was provided in the unsafe area (due to the circumstances explained above)	No, medical care was provided on scene with police forces securing the zone
Have there been any delays in medical care due to safety issues such as threats to rescuers?	no	no
If so, how long did it take until the last casualty had received medical care?		
Δ t from first emergency call until transport of the first patient with triage category T1/RED to hospital	Δ t: 38 min	Δ t: 22 min
Δ t from first emergency call until transport of the first patient with triage category T2/YELLOW to hospital	Δ t:41 min	Not applicabe
Δ t from first emergency call until transport of the first patient with triage category T3/GREEN to hospital	unknown	Δ t: 64 min
Δ t from first emergency call until arrival of the first patient with category T1/RED in hospital	Δ t: 48 min	Δ t: 28 min
Δ t from first emergency call until arrival of the first patient with category T2/YELLOW in hospital	unknown	Not applicable
Δ t from first emergency call until arrival of the first patient with category T3/GREEN in hospital	Δ t: 77 min	Δ t: 68 min (estimation)
Δ t from first emergency call until transport of the last patient with categoryT1/ RED to hospital	Δ t: 80 min	Δ t: 49 min
Δ t from first emergency call until transport of the last patient with category T2/YELLOW to hospital	none	Not applicable
Δ t from first emergency call until transport of the last patient with category T3/GREEN to hospital	none	Not applicable
Δ t from first emergency call until arrival of the last patient with category T1/RED in hospital	Δ t: 79 min	Δ t: 55 min (estimation)
Δ t from first emergency call until arrival of the last patient with category T2/YELLOW in hospital	none	Not applicable
Δ t from first emergency call until arrival of the last patient with category T3/GREEN in hospital	none	Not applicable
Δ t from first emergency call until last patient found	Δ t:36 min	unknown
Δ t from first emergency call until identification of all patients	Δ t:106 min	unknown

**Table 6:** Hospitals.

Hospitals	Incident 2016	Incident 2021
Δ t from first notification/alerting of the hospitals until arrival of the first patient	Hospital 1: Δ t = 36 min Hospital 2: Δ t = 36 min Hospital 3: Δ t = 36 min	Hospital 1: Δ t = 10 min Hospital 2: Δ t = unknown Hospital 3: Δ t = unknown
Do the hospitals have emergency alarm and response plans (HEARP – hospital emergency alarm and response plan)?	Hospital 1: yes Hospital 2: yes Hospital 3: yes	Hospital 1: yes Hospital 2: yes Hospital 3: yes
Have they (HEARP) been activated?	Hospital 1: no Hospital 2: no Hospital 3: no	Hospital 1: yes Hospital 2: yes Hospital 3: no
Have they (HEARP) been successfully applied?	not applicable	
When was the dispatch centre informed about the capacity of the hospitals to receive and treat casualties? Δ t from first notification until capacity information	Hospital 1: 6 min Hospital 2: 5 min Hospital 3: 5 min	Hospital 1: 0 min Hospital 2: 3 min Hospital 3: unknown
Ratio between announced vs actually delivered causalities	Hospital 1: 4/4 Hospital 2: 1/1 Hospital 3: 1/1	Hospital 1: 5/5 Hospital 2: 2/2 Hospital 3: 1/1
Adequate allocation and distribution of causalities?	yes	yes
Number of self-referred patients	zero	zero
Did the hospitals have a strategy in place to deal with terroristic mass casualties?	Hospital 1: no Hospital 2: no Hospital 3: no	Hospital 1: yes Hospital 2: yes Hospital 3: yes

**Table 7:** Emergency psycho-social support (EPSS).

Psychosocial emergency relief	Incident 2016	Incident 2021
Δ t from first establishment of the situation to information relay to the psychosocial emergency services	Δ t: 14 min	Δ t: 7 min
Has the support through police and medical services been coordinated?	yes	yes
Have EPSS points of contacts been established during the days after the incident?	Yes	yes
Has EPSS been offered to uninjured persons	yes	yes
Has EPSS ben offered to the rescue and police forces	yes	yes
Was there a felt/real threat to the rescue forces at any point?	Yes	yes
How many casualties received EPSS?	none	none
How many of the uninjured affected persons received EPSS?	73	44
How many of the police forces received EPSS?	unknown	130
How many of the rescue forces received EPSS?	67	

A summary of the lessons identified evaluation is given in Table 8.

**Table 8:** Summary of the results.

Lessons	Content	Result
LI 2016–1 + 2	Response plan for MASCAL and terroristic attack in place?	LL 2016/2021
LI 2016–3	High priority communication	LI 2016/2021
LI 2016–4	Zoning	LI 2016/2021
LI2016-5	Mutal exchange of strategies and tactics	LI 2016/2021
LI 2016–6	Command and Control structures	LL 2016/2021
LI 2016–7	Communication infrastructure	LL 2016/2021
LI 2016–8	Common situational picture	LL 2016/2021
LI 2016–9	Involvement of hospitals	LL 2016/2021
LI 2016–10	Emergency Psycho-Social Support (EPSS)	LL 2016/2021

*LI* lesson identified, *LL* lesson learned, *MASCAL* mass casualty.

The lessons from 2016 and their evaluation results are now presented below.5

### LI 2016–1 + 2

A general response plan for mass casualty situations is an essential basis for successful response (LI number 1) and serves as a basis for a dedicated response plan for rampage and terror attacks (LI number 2). Both concepts must be trained and practiced in order to apply them effectively during a mission. Drils will be held once a year.

### Result

The Bavarian response plan to terroristic attacks was well established, trained and applied by the medical emergency services. Therefore the LI 2016–1 + 2 were proofed as LL 2016/2021.

### LI 2016–3

Establishment of a high priority communication infrastructure between the police dispatch centre and the medical rescue dispatch centre (“red phone”) is a crucial point. This is vital in order to provide a constant flow of information between a defined dispatcher and recipient. This communication is essential not only during the initial phase (early and fast track evaluation of the situation via this communication channel) but throughout the mission. The aim is to provide continuity of information between exactly defined individuals in order to allow information gathering instead of information dispersal.

### Result

The red phone as a high priority communication infrastructure between the police dispatch center and the medical rescue control center was established and applied. But staffing and operating was almost impossible during the initial „chaos phase “. The further evaluation revealed that the amount of information in the initial phase was too much for one person to handle. The lesson was thus classified as LI 2016/2021.

As a consequence, it was concluded that only a small amount of highly relevant information should be exchanged in the initial phase. These components were defined as threat, casualties zoning and deployed forces.

### LI 2016–4

A commonly (police and EMS) decided and consented organization of the scene and zoning was identified as a crucial point for the successful completion of the mission.

- Establishment of a common access route to the incident area used by police, firearm services and emergency medical services.
- Establishment of a common area zoning, especially a common and safe deployment sector.
- Establishing of a common on-site command post.
- The incident area should be divided into a red (unsafe), yellow (secured) – green (safe) zone with different modes of action within these zones.

### Result

In the initial phase of a terror or amok rescue mission, a green/safe zone cannot be determined by the police with sufficient certainty. Especially in the early phase, the situation is dynamic, the information is contradictory and neither other perpetrators nor the risk of a second hit can be ruled out with certainty. Accordingly, police leaders are not able to define a safe zone with sufficient certainty. The lesson was classified as LI 2016/2021. As a consequence it was concluded that restricting the treatment of casualties by medical rescue teams to a safe area would lead to a poorer outcome. It is a strategic goal to secure the incident site with police forces in such a way that the unarmed rescue teams can treat and evacuate the casualties at an acceptable risk. The lesson was thus classified as LI 2016/2021.

### LI2016-5

Strategies and tactics need to be developed and practiced beforehand. Without instructions, knowledge and specific training none of the strategies can be used on scene in a life-threatening situation.

We have formulated the following key points to prepare the rescue forces for future incidents:

- Are there comprehensive management plans in place?
- Are these plans well known and well trained?
- Are there common drill and training possibilities?
- Are there clear mission goals and strategies?
- Is there stockpiling of the equipment and material?
- Is this equipment and material readily available?

### Result

The strategies and tactics were well known and trained by the police forces and the rescue services. However, the strategies and tactics of the rescue services were not sufficiently known by the police forces and, conversely, the police strategies and tactics were not sufficiently known by the emergency rescue services. Therefore the lesson was classified as LI 2016/2021. It was concluded, that a mutual exchange and a joint preparation is essential and will be established.

### LI2016-6

Early establishment of a command and control structure is crucial. However, autonomous management of dedicated sections is essential in highly dynamic situations.

### Result

The command and control structure was established early in the mission course, therefore this lesson was classified as LL 2016/2021.

### LI2016-7

A predefined and common communication infrastructure for all operational forces is a crucial point for the successful completion of the mission.

### Result

The communication infrastructure was well established and applied successfully during the mission. Therefore this lesson was classified as LL 2016/2021.

### LI2016-8

A successful mission response requires continuous flux of reports the on-site command post. This includes “up to date” situational information from all different sub sectors of the mission as well as reliable information about the safety situation at the incident site.

### Result

There was a continuous flux of reports and the on-site commander had a common Therefore the 1_LI 2016 was proofed as LL 2016/2021.

### LI2016-9

Hospitals play a vital role in the treatment of patients in the case of terrorist attacks. They are a key element in order to evacuate patients from an unsafe environment towards definitive medical treatment. Therefore hospitals need to be involved in the medical response from the very beginning.

### Result

The regional hospitals were alerted early and were ready to receive patients at any time. The red triaged patients were transported to the University level 1 trauma center at short intervals. Admission to the hospitals went without a problem. The lesson was classified as LL 2016/2021.

### LI2016-10

The organization of Emergency Psycho-Social Support (EPSS) is of great importance for the management of a terroristic attack. This involves early intervention on the day of the incident and processing in the period that follows. EPSS must be provided to the victims and to the rescue forces as well.

### Result

After the operation, there were EPSS offers for emergency services and fire brigades. EPSS options for victims, witnesses and residents were offered in cooperation with the police forces. The measure deemed suitable, the integration of EPSS into the response plans was successful. EPSS was well integrated into operations and command structures. Furthermore, the staffing of the EPSS centre and the cooperation with the police, including the joint delivery of fatal outcome communication worked well. There was also consistent EPSS directed at the emergency services with debriefings in a shielded area afterwards. According to the result, the lesson identified was classified as a LL2016/21.

## Discussion

In a recent systematic review, we identified lessons learned after terror attacks during the last 20 years, but often knowledge gained have not yet been implemented into routine, thus the so-called lessons learned were in fact lessons identified. In this respect, the NATO recently recommended that lessons learned is the end point of a three-step process, beginning with identification of lessons, assigning them to an appropriate action, training the staff closing the learning circle after checking the successful implementation. In our study, we classified seven true lessons learned, as they were included in the response plans, were properly applied during the mission and proofed to be appropriate with regard to tactical issues. However, three lessons were applied during the operation but didn´t work well, so they were categorized as lessons identified (communication, zoning and the mutual exchange of different tactical approaches).

As a result, these new lessons identified were selected and solutions were developed in working groups. This led to an adaptation of the emergency plans plans and the tactical approach. The learning process for the LI 2016/2021 has been almost completed, while the other two LI still need to be finalised. In any case, the intensive work has led to a better exchange and mutual understanding between the involved police and rescue forces. This effect in itself is extremely valuable.

Communication difficulties and the challenge to share and distribute high relevant information to the rescue forces on scene and back to the dispatch centers during the initial phase of a major incident have been described^13,16–19^. Schorscher et al. published a systematic review on lessons learned from terror attacks (2001–2017) and communication was identified as one of the unresolved problems since 2001^13^. De Cauwer et al. reported different types of communication failures, while “nor or inadequate communication as well as safety concerns for EMS personal” were predominant”^19^. Other important reasons for communication problems were the failure of mobile phone and EMS radio network and the lack of training in using the radio^19^. These difficulties were also reported by Hansen et al.^20^. The evaluation of a mass shooting event in Denmark revealed a failure to use the predefined communication channels in more than one third of the rescue forces. The lack of basic training and unintuitive interface design were mentioned as the main reasons for this observed facts. In the first incident in 2016, we identified an insufficient communication structure and a lack of information between police forces and rescue forces. Therefore, an exclusive telephone-hotline was established between the two dispatch centers. Five years later the hotline was used intensively, but the huge amount of information was uncontrolled and overwhelmed the communication officers. Now, we concluded that early communication is vital, but that the content must be limited to the most important information. Lapostolle et al. address the early chaos phase of a terrorist attack and emphasize, that simple and clear communication is essential at this stage^21^. The 3-Echo concept, published by Autrey et al. defined the reporting of hazards and casualties, staging locations and incident talk group assignments as the four most important elements of early communication^1^. In our expert group we proposed four items of highlighted importance at the early stage of terror response: Threat, Zoning, Casualties and deployed forces. The National Fire Protection Association (USA) has released a standard for active shooter/hostile events (ASHE)^22^. For the incident size- up, they define 7 important elements: The major incident notification, the specific location and characteristics, the type of incident, the hazards and the number of potential assailants, the access and staging of incoming units, the approximate number of victims and additional resources needed. Although three more items are used in this concept, the main information corresponds to those defined in our concept.

Access to wounded casualties and emergency care is a major issue during terroristic attacks, especially in dynamic and uncontrolled situations. The focus of the police forces is to stop the killing and in most systems word wide the emergency medical service is not trained and equipped to act in an uncontrolled threatening environment (red zone)^6^. Although a growing number of police forces are trained in basic emergency interventions (e.g. stop the bleeding) their mission focuses on controlling the threat^6^. This therapeutic vacuum in the red zone causes a delay in emergency care for the wounded casualties, which is associated to higher rates of fatal outcome^6^. There are different approaches to address this problem. In France, there are specially trained emergency physicians who operate in the red zone together with special police forces. During the terrorist attack in Paris in November 2015 doctors of the RAID (Recherche, Assistence, Intervention, Dissuassion) were deployed in the hot zone and saved lives under the direct threat of the terrorists^6^. Park et. Al describes a time gain of more than 3 h by starting professional medical care in the hot zone^6^. Joint training and the direct integration of the doctors into the special forces team made this approach possible^6^. In Denmark tactical emergency casualty care (TECC®) is provided by tactical emergency medical service (TEMS). Paramedics and emergency physicians are trained and equipped to act in an unsafe environment in order to stop the dying at a very early stage^20^. This concept has been introduced in 2018 in the capital region of Denmark and the TEMS unit is dispatched approximately 200 times a year^20^.

In Germany, the nationwide consent proposed a three zones model (red/unsafe, yellow/semi-safe and green/safe)^23^. Rescue forces are generally not deployed in the red zone and most of the concepts provide for initial casualty care in the green zone. In 2016 we concluded, that initial zoning in red, yellow and green zones by the police forces is a fundamental step in order to organize the mission and to safely deploy the rescue forces. In our 2021 evaluation, we found that early establishment of a green zone in the initial phase of a terrorist attack was not feasible with sufficient certainty. Especially in the early mission stages, the situation is dynamic, the available information is contradictory and neither other perpetrators nor the risk of a second hit can be ruled out with certainty. Accordingly, police leaders are not able to define a green zone at that early time. To avoid a delay of medical treatment and to deploy rescue forces with an acceptable risk we suggest to provide casualty care in a yellow zone. As soon as police forces are able to secure certain areas or corridors, these are defined as a yellow zone, where rescue forces are allowed to provide initial lifesaving casualty care.

Protected casualty collection points may serve as hand over point from hot zone to yellow zone. In order to clear up the scene immediately treatment time in the yellow zone should be restricted to a minimum. Both, the 3 Echo concept and the THREAT concept focus on rapid evacuation of casualties along secured corridors^1^^,^^24^. In the standard for active shooter/hostile events (ASHE)^22^ the fire brigades and EMS are enabled to act in a warm (yellow) zone in order to provide threat based triage and care. Fire and EMS personal are not allowed to act in the hot (red) zone without being part of the special forces^22^.

Cooperation and multidisciplinary approach is a crucial point for effective response to terroristic attacks^13^. Intense collaboration of the military, police forces, fire brigades and the EMS was identified as a very important lesson learnt in a systematic review published by Shorscher et al. An important step to improve interdisciplinary cooperation is joint training and drills on a local, regional and national level^25^.

It is very important for improvement at national and international level to learn from each other and to report events in a standardized way. The major incident reporting web is particularly worth mentioning here, where a template of data is collected and published, making the operations transparent and usable for everyone^26^**.**

We now found that the strategies and tactics of the rescue services were not sufficiently known by the police forces and, conversely, the police strategies and tactics were not sufficiently known by the emergency rescue services, although this issue has been identified after the 2016 attack. The ASHER program emphasizes a whole community preparedness that enables an integrated response including the police units, the emergency services, and other organizations such as health care facilities, the public health service, administrations, political decision-makers, local businesses and the local authorities^22^. This holistic approach is probably the concept of the future.

## Limitations

A possible limitation is that we have not chosen a formal but a group cerntered consesus process. In formulating the results, we relied on the discussion of the expert panel. However, there were no controversial decisions here, so that a formal consensus process would not have lead to different results.

## Conclusion

Lessons learned are usually defined during the evaluation and assessment of rescue missions, irrespective whether they have been really learned or only identified. Here, we evaluated two major incidents regarding lessons identified or learned according to the NATO recommendations. This procedure is vital for the continuous improvement of response plans to disasters and emergencies in future.

## References

[1] Autrey, A. W. et al. 3 Echo: concept of operations for early care and evacuation of victims of mass violence. *Prehosp. Disaster Med. ***29**(4), 421–428 (2014). 24909363 10.1017/S1049023X14000557

[2] Hirsch, M. et al. The medical response to multisite terrorist attacks in Paris. *Lancet ***386**(10012), 2535–2538 (2015). 26628327 10.1016/S0140-6736(15)01063-6

[3] Ghanchi, A. Insights into French emergency planning, response, and resilience procedures from a hospital managerial perspective following the Paris terrorist attacks of friday, november 13, 2015. *Disaster Med. Public Health Prep. ***10**(5), 789–794 (2016). 27775505 10.1017/dmp.2016.21

[4] Borel, M. et al. Organization in response to massive afflux of war victims in civilian practice—experimental feedback from the November 2015 Paris terrorist attacks. *J. Visc. Surg. ***154**(Suppl 1), S3-s7 (2017). 29055662 10.1016/j.jviscsurg.2017.07.007

[5] Wurmb, T. et al. Structured analysis, evaluation and report of the emergency response to a terrorist attack in Wuerzburg, Germany using a new template of standardised quality indicators. *Scand. J. Trauma, Resusc. Emerg. Med. ***26**(1), 87 (2018). 30340516 10.1186/s13049-018-0555-5 PMC6194622

[6] Park, C. L. et al. How to stop the dying, as well as the killing, in a terrorist attack. *Bmj ***368**, m298 (2020). 32001528 10.1136/bmj.m298

[7] Wurmb, T. H. & Hossfeld, M. Notfallmedizinische Versorgung bei Terror- und Amoklagen. *Refresher Course - Aktuelles Wissen für Anästhesisten*10.1055/s-0042-120229 (2018).

[8] Hossfeld, B. et al. Massenanfall von Verletzten – Besonderheiten von bedrohlichen Lage. *Ains Anästhesiologie Intensivmedizin ***52**, 618–629 (2017). 10.1055/s-0042-120229 28886611

[9] Wurmb, T. et al. Bewältigung von besonderen Bedrohungslagen. *Notfall + Rettungsmedizin ***21**(8), 66–672 (2018).

[10] Wurmb, T., Hossfeld, B. & Zoller, G. Polizei und Rettungsdienst bei der Bewältigung lebensbedrohlicher Einsatzlagen. *Notfall + Rettungsmedizin. ***21**(7), 57–584 (2018).

[11] Philippe, J.-M. et al. French Ministry of Health’s response to Paris attacks of 13 November 2015. *Crit. Care ***20**(1), 85 (2016). 27039082 10.1186/s13054-016-1259-8 PMC4818525

[12] Goralnick, E., Van Trimpont, F. & Carli, P. Preparing for the next terrorism attack: lessons from Paris, brussels, and Boston. *JAMA Surgery ***152**(5), 419–420 (2017). 28122085 10.1001/jamasurg.2016.4990

[13] Schorscher, N. et al. Lessons learned from terror attacks: thematic priorities and development since 2001-results from a systematic review. *Eur. J. Trauma Emerg. Surg.*10.1007/s00068-021-01858-y (2022). 35024874 10.1007/s00068-021-01858-y PMC8757406

[14] NATO, J.A.a.L.L.C., *The NATO Lessons Learned Handbook 4th edition.* 2022.

[15] Wurmb, T. et al. Quality indicators for rescue operations in terrorist attacks or other threats: A pilot study after the Wurzburg terrorist attack of July 2016. *Anaesthesist ***66**(6), 404–411 (2017). 28386683 10.1007/s00101-017-0298-0

[16] Aylwin, C. J. et al. Reduction in critical mortality in urban mass casualty incidents: analysis of triage, surge, and resource use after the London bombings on July 7, 2005. *Lancet ***368**(9554), 2219–2225 (2006). 17189033 10.1016/S0140-6736(06)69896-6

[17] Hunt, P. Lessons identified from the 2017 Manchester and London terrorism incidents. Part 1: introduction and the prehospital phase. *BMJ Military Health ***166**(2), 111 (2020). 29653938 10.1136/jramc-2018-000934

[18] Lockey, D. J. et al. London bombings July 2005: the immediate pre-hospital medical response. *Resuscitation ***66**(2), ix–xii (2005). 16053939 10.1016/j.resuscitation.2005.07.005

[19] De Cauwer, H. et al. Communication failure in the prehospital response to major terrorist attacks: lessons learned and future directions. *Eur. J. Trauma. Emerg. Surg. ***49**(4), 1741–1750 (2023). 36214838 10.1007/s00068-022-02131-6

[20] Hansen, P. M. et al. The Field’s mass shooting: emergency medical services response. *Scand. J. Trauma, Resusc. Emerg. Med. ***31**(1), 71 (2023). 37919753 10.1186/s13049-023-01140-7 PMC10621148

[21] Lapostolle, F. et al. Comment appréhender une tuerie de masse pour les équipes Smur primo-intervenantes ?. *Annales françaises de médecine d’urgence*10.3166/afmu-2018-0084 (2018).

[22] (NFPA), T.N.F.P.A. *NFPA 3000®: STANDARD FOR AN ACTIVE SHOOTER/HOSTILE EVENT RESPONSE (ASHER) PROGRAM*. 2024; Available from: https://www.nfpa.org/for-professionals/codes-and-standards/nfpa-link). .

[23] Wurmb, T. et al. Emergency response to terrorist attacks: results of the federal-conducted evaluation process in Germany. *Eur. J. Trauma Emerg. Surg. ***46**(4), 725–730 (2020). 32206880 10.1007/s00068-020-01347-8 PMC7429537

[24] Jacobs, L. M. et al. The Hartford consensus: THREAT, a medical disaster preparedness concept. *J. Am. Coll. Surg. ***217**(5), 947–953 (2013). 24139220 10.1016/j.jamcollsurg.2013.07.002

[25] Schorscher, N. Systematic literature review on lessons learnt from terrorist attacks with a focus on pre-hospital and in-hospital management. *Klinik und Poliklinik für Anästhesiologie, Intensivmedizin, Notfallmedizin und Schmerztherapie der Universitätsklinik Würzburg ***9**, 93–95 (2022).

[26] Hardy, S. E. J. & Fattah, S. Trials and tribulations: how we established a major incident database. *Scand. J. Trauma, Resusc. Emerg. Med. ***25**, 7. 10.1186/s13049-017-0351-7 (2017). 28122602 10.1186/s13049-017-0351-7 PMC5267420

